# Ancient and Recent Selective Pressures Shaped Genetic Diversity at AIM2-Like Nucleic Acid Sensors

**DOI:** 10.1093/gbe/evu066

**Published:** 2014-03-28

**Authors:** Rachele Cagliani, Diego Forni, Mara Biasin, Manuel Comabella, Franca R. Guerini, Stefania Riva, Uberto Pozzoli, Cristina Agliardi, Domenico Caputo, Sunny Malhotra, Xavier Montalban, Nereo Bresolin, Mario Clerici, Manuela Sironi

**Affiliations:** ^1^Bioinformatics Laboratory, Scientific Institute IRCCS E. Medea, Bosisio Parini (LC), Italy; ^2^Department of Biomedical and Clinical Sciences, University of Milan, Italy; ^3^Department of Neurology-Neuroimmunology, Centre d’Esclerosi Múltiple de Catalunya, Cemcat, Hospital Universitari Vall d'Hebron (HUVH), Barcelona, Spain; ^4^Laboratory of Molecular Medicine, Don C. Gnocchi Foundation ONLUS, IRCCS, Milan, Italy; ^5^Dino Ferrari Centre, Department of Physiopathology and Transplantation, University of Milan, Fondazione Ca' Granda IRCCS Ospedale Maggiore Policlinico, Milan, Italy; ^6^Chair of Immunology, Department of Physiopathology and Transplantation, University of Milan, Italy

**Keywords:** AIM2-like receptors, positive selection, long-standing balancing selection, IFI16

## Abstract

AIM2-like receptors (ALRs) are a family of nucleic acid sensors essential for innate immune responses against viruses and bacteria. We performed an evolutionary analysis of ALR genes (*MNDA, PYHIN1, IFI16*, and *AIM2*) by analyzing inter- and intraspecies diversity. Maximum-likelihood analyses indicated that *IFI16* and *AIM2* evolved adaptively in primates, with branch-specific selection at the catarrhini lineage for *IFI16*. Application of a population genetics–phylogenetics approach also allowed identification of positive selection events in the human lineage. Positive selection in primates targeted sites located at the DNA-binding interface in both IFI16 and AIM2. In IFI16, several sites positively selected in primates and in the human lineage were located in the PYD domain, which is involved in protein–protein interaction and is bound by a human cytomegalovirus immune evasion protein. Finally, positive selection was found to target nuclear localization signals in IFI16 and the spacer region separating the two HIN domains. Population genetic analysis in humans revealed that an *IFI16* genic region has been a target of long-standing balancing selection, possibly acting on two nonsynonymous polymorphisms located in the spacer region. Data herein indicate that ALRs have been repeatedly targeted by natural selection. The balancing selection region in *IFI16* carries a variant with opposite risk effect for distinct autoimmune diseases, suggesting antagonistic pleiotropy. We propose that the underlying scenario is the result of an ancestral and still ongoing host–pathogen arms race and that the maintenance of susceptibility alleles for autoimmune diseases at *IFI16* represents an evolutionary trade-off.

## Introduction

Mammalian nucleic acid-sensing receptors play essential roles in the recognition of infectious agents and in triggering innate and adaptive immune responses. Different classes of nucleic acid-sensing molecules have been identified; these molecules are classified on the basis of cellular localization, target specificity, and downstream signaling pathway. Among them, toll-like receptors (TLRs) are the best characterized class, and at least four members (TLR3, TLR7, TLR8, and TLR9), located at the endosomal membrane, are specialized in viral sensing (Desmet and Ishii 2012). TLRs signal through MyD88 or TRIF to induce the release either of inflammatory cytokines or of type I interferons (IFNs). The cytoplasmic detection of viral RNA is also mediated by cytosolic RIG-I-like receptors (RIG-I, IFIH1, and DHX58), which elicit type I IFN responses through the mitochondrial antiviral signaling (MAVS) adaptor (Desmet and Ishii 2012). NOD2, a member of the NOD-like receptor (NLR) family, also impinges on MAVS upon sensing single-stranded RNA (ssRNA), whereas NLRP3 is activated by ssRNA or dsRNA resulting in the formation of the inflammasome complex, which is mediated by PYCARD (also known as ASC) (Desmet and Ishii 2012). Finally, the IFN-inducible HIN-200 gene family, also called PYHIN gene family, comprises a class of homologous viral sensor proteins characterized by the presence of an N-terminal pyrin-domain and a 200-amino acid signature motif (HIN-200 domain) (Veeranki and Choubey 2012).

**Fig. 1.—:**
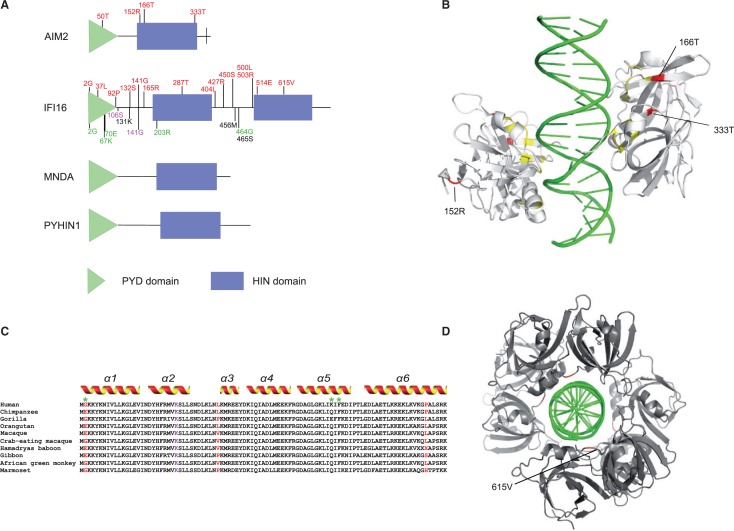
ALR gene domain representation and analysis of positively selected sites. (*A*) Domain structure of the four ALR genes. Positively selected sites in whole phylogeny (identified through both BEB and MEME) are shown in red; residues subject to positive selection in the human (green) and in the catarrhini (black: BEB sites; magenta: BEB and MEME sites) lineages are also indicated. (*B*) Three-dimensional (3D) structure of the AIM2 HIN:DNA complex (pdb ID 3RN2). Positively selected sites are shown in red, and yellow indicates key residues at the HIN:DNA interface. Positions refer to the human sequence. (*C*) Alignment of the IFI16 PYD domain. Positively selected sites in whole phylogeny are shown in red, whereas human-specific positively selected sites are marked by a green asterisks. A lysine residue conserved among PYD domains from different proteins is shown in violet. The extension of the six α-helices, inferred from structure analysis of human PYDs, is shown. (*D*) 3D structure of the IFI16 HIN:DNA complex (pdb ID 3RNU). The 615T positively selected site (red) is located at the DNA-binding interface formed by the 4 IFI16 monomers.

In humans, four members of this family have been identified and are encoded by a cluster of genes (*MNDA*, *PYHIN1*, *IFI16*, and *AIM2*) located on chromosome 1. These proteins share a pyrin motif involved in protein–protein interactions, as well as one (AIM2, MNDA, and PYHIN1) or two (IFI16) HIN-200 domains that mediate binding to double-stranded DNA (dsDNA) (fig. 1) (Schattgen and Fitzgerald 2011). The best studied PYHIN family members are AIM2 and IFI16. The former is a sensor of cytosolic DNA, which triggers the inflammosome pathway through PYCARD resulting in caspase-1-mediated cleavage of IL-1β (Schattgen and Fitzgerald 2011). Conversely, IFI16 has a mainly nuclear activity (in analogy to MNDA and PYHIN1), although it can also sense dsDNA in the cytoplasm, as its nuclear-cytoplasmic shuttling is regulated by a multipartite nuclear localization signal (Li et al. 2012). IFI16 signals through STING-dependent pathways. PYHIN proteins, according to their function as innate DNA sensors, are also termed AIM2-like receptors (ALRs) (Unterholzner et al. 2010).

A recent analysis of ALR genes in mammals indicated that the cluster is extremely dynamic: Distinct species carry diverse sets of functional genes, suggesting that strong selective pressures have been acting on these loci (Brunette et al. 2012). Indeed, evolutionary analysis of other genes involved in nucleic acid sensing or in the downstream signaling pathways identified signatures of natural selection. Thus, *MAVS* evolved adaptively in primates, the underlying pressure being accounted for by hepaciviruses (Patel et al. 2012). Likewise, analysis of RIG-I-like receptors in human populations revealed signatures of local adaptation at the *IFIH1* and *DHX58* genes (Fumagalli et al. 2010; Vasseur et al. 2011; Quintana-Murci and Clark 2013). These observations are in line with viruses, and pathogens, in general, being a major determinant of molecular evolution in mammals and human populations (Kosiol et al. 2008; Fumagalli et al. 2011). Herein, we performed an evolutionary study of the ALR cluster by analyzing both inter- and intraspecies diversity.

## Materials and Methods

### Evolutionary Analysis in Mammals

Primate sequences for *MNDA*, *PYHIN1*, *IFI16,* and *AIM2* were retrieved from the Ensembl website (http://www.ensembl.org/index.html, last accessed January 30, 2014) and National Center for Biotechnology Information (NCBI) database (http://www.ncbi.nlm.nih.gov/, last accessed January 30, 2014). *IFI16* coding sequencing information for *Macaca fascicularis* and *Chlorocebus aethiops* were obtained by real time polymerase chain reaction (PCR) amplification of RNA derived from CYNOM-K1 and COS1 cells. Primer sequences are available in supplementary table S1, Supplementary Material online. The species list for all genes is reported in supplementary table S2, Supplementary Material online.

DNA alignments were performed using the RevTrans 2.0 utility (Wernersson and Pedersen 2003), which uses the protein sequence alignment as a scaffold for constructing the corresponding DNA multiple alignment. This latter was checked and edited by hand to remove alignment uncertainties. For PAML analyses (Yang 2007), we used trees generated by maximum likelihood (ML) using the program PhyML (Guindon et al. 2009).

To detect selection, NSsite models that allow (M8, M2a) or disallow (M1a and M7) sites to evolve with d*N*/d*S* > 1 were fitted to the data using the F3x4 and F61 codon frequency model. Whenever ML trees showed differences (always minor) from the accepted primate phylogeny, analyses were repeated using the accepted tree, and the same results were obtained in all cases. Sites under selection with the M8 model were identified using Bayes empirical Bayes (BEB) analysis using a significance cutoff of 0.90 (Anisimova et al. 2002; Yang et al. 2005).

To explore possible variations in selective pressure among different lineages, we applied the free-ratio models implemented in the PAML package: The M0 model assumes all branches to have the same ω, whereas M1 allows each branch to have its own ω (Yang and Nielsen 1998). The models are compared through likelihood-ratio tests (degree of freedom = total number of branches − 1). To identify specific branches with a proportion of sites evolving with ω > 1, we used branch-site-random effects likelihood (BS-REL; Kosakovsky Pond et al. 2011). Branches identified using this approach were cross-validated with the branch-site likelihood ratio tests (LRTs) from PAML (the so-called modified model A and model MA1, “test 2”) (Zhang et al. 2005). The advantage of this method is that it also implements a BEB analysis analogous to that described earlier to calculate the posterior probabilities that each site belongs to the site class of positive selection on the foreground lineages. Thus, BEB allows identification of specific sites that evolve under positive selection on specific lineages, although it has limited statistical power (Zhang et al. 2005).

Genetic Algorithm Recombination Detection (GARD; Kosakovsky Pond et al. 2006), mixed effects model of evolution (MEME; Murrell et al. 2012), single likelihood ancestor counting (SLAC; Kosakovsky Pond and Frost 2005), and BS-REL analyses were performed through the DataMonkey server (Delport et al. 2010) (http://www.datamonkey.org, last accessed January 30, 2014).

### Population Genetics–Phylogenetics Analysis

Data from the Pilot 1 phase of the 1000 Genomes Project were retrieved from the dedicated website (1000 Genomes Project Consortium et al. 2010). Low-coverage single-nucleotide polymorphism genotypes were organized in a MySQL database. A set of programs was developed to retrieve genotypes from the database and to analyze them according to selected regions/populations. These programs were developed in C++ using the GeCo++ (Cereda et al. 2011), the libsequence (Thornton 2003), and the mysqlpp libraries. Coding sequence information was obtained for the four ALR genes. To analyze the distribution of fitness effects (DFEs) for ALR genes, we used gammaMap (Wilson et al. 2011). We assumed θ (neutral mutation rate per site), *k* (transitions/transversions ratio), and *T* (branch length) to vary among genes following log-normal distributions. For each gene, we set the neutral frequencies of non-STOP codons (1/61) and the probability that adjacent codons share the same selection coefficient (*P* = 0.02). For selection coefficients, we considered a uniform Dirichlet distribution with the same prior weight for each selection class. For each gene, we run 10,000 iterations with a thinning interval of 10 iterations.

### HapMap Samples and Sequencing

Human genomic DNA from 60 HapMap subjects (20 individuals for Yoruba [YRI], 20 Europeans [CEU], and 20 East Asian [AS] subjects) was obtained from the Coriell Institute for Medical Research (http://www.coriell.org/, last accessed January 30, 2014). The analyzed region (NCBI/hg18 chr1:157267539–157272405) was PCR amplified and directly sequenced. PCR products were treated with ExoSAP-IT (USB Corporation, Cleveland, OH), directly sequenced on both strands with a Big Dye Terminator sequencing Kit (v3.1, Life Technologies), and run on an Applied Biosystems ABI 3130 XL Genetic Analyzer (Life Technologies). Sequences were assembled using AutoAssembler version 1.4.0 (Life Technologies) and inspected manually by two distinct operators. Primer sequences are available in supplementary table S1, Supplementary Material online.

### Primate Samples

The CYNOM-K1 and COS1 cells, as well as genomic DNA from *Gorilla gorilla* and *Pongo pygmaeus,* were obtained by the European Collection of Cell Cultures. The genomic DNA of eight *Pan troglodytes* was kindly provided by the Gene Bank of Primates, Primate Genetics, Germany (http://dpz.eu/index.php, last accessed January 30, 2014). These samples belong to the *Pan troglodytes verus* subspecies (Cagliani et al. 2012). The IFI16 coding sequences for *M. fascicularis* and *C. aethiops* have been submitted to GenBank (provisional IDs: KF154419 and KF154420).

### Segmental Duplication Genotyping

The segmental duplication of exon 7 in *IFI16* was analyzed using a PCR-based method. In particular, PCR amplifications were performed with JumpStart AccuTaq LA DNA Polymerase (Sigma-Aldrich) and two sets of primers: one that amplifies only the duplicated form (F: GTCCTGTGCACCTTGTGTCA; R: CTGATGTATGGTGAGAGAGC) and one that flanks the segmental duplication (F: GTCCATTTCTGTAGCCATAGG; R: TCTGAGTTGTAGGAGAGCACT). The PCR products were electrophoretically separated on agarose gels.

### *F*_ST_** Analysis

Human Genome Diversity Project (HGDP CEPH) panel data derive from a previous work (Li et al. 2008). Atypical or duplicated samples and pairs of close relatives were removed (Rosenberg 2006). Following previous indications (Fumagalli et al. 2009a, 2009b), Bantu individuals (South Africa) were considered as one population. *F*_ST_ was calculated for all HGDP-CEPH variants among continental groups using the R package HIERFSTAT (Goudet 2005); *F*_ST_ distributions were calculated after binning single-nucleotide polymorphisms (SNPs) into minor allele frequency (MAF) classes (50 quantile classes based on MAF calculated over the whole panel); outliers are defined as variants with an *F*_ST_ higher than the 95th percentile in the distribution of SNPs in the same MAF class.

As for the 1000 Genomes Project data, genotype information was obtained for the analyzed genomic region (NCBI/hg18, chr1:157063927–157317926) and for 2,000 randomly selected RefSeq genes. *F*_ST_ sliding window analysis was performed on overlapping 20 SNP windows moving with a step of three SNPs. The numbers of windows used to obtain a reference distribution (i.e., deriving from the 2,000 randomly selected genes) were 120,978 (YRI/CEU), 111,227 (YRY/AS), and 81,557 (CEU/AS).

### Population Genetic Analyses

Tajima's *D* (Tajima 1989), Fu and Li's D* and F* (Fu and Li 1993) statistics, and diversity parameters θ_W_ (Watterson 1975) and π (Nei and Li 1979) were calculated using libsequence (Thornton 2003). Calibrated coalescent simulations were performed using the cosi package (Schaffner et al. 2005) and its best-fit parameters for YRI, CEU, and AS populations with 10,000 iterations. Coalescent simulations were conditioned on mutation rate, and recombination rate was derived from UCSC tables (http://genome.ucsc.edu/, last accessed January 30, 2014, snpRecombRateHamap table).

The ML-ratio Hudson, Kreitman, and Aguadé (HKA) test was performed using the MLHKA software (Schaffner et al. 2005), as previously proposed (Fumagalli et al. 2009a). For human populations, 99 reference loci were randomly selected among National Institute of Environmental Health Sciences (NIEHS) loci that have been resequenced in YRI, CEU, and AS. Genotype data for 5-kb regions from 238 resequenced human genes were derived from the NIEHS SNPs Program web site (http://egp.gs.washington.edu, last accessed January 30, 2014). In particular, we only selected genes that had been resequenced in populations of defined ethnicity including YRI, CEU, and AS (NIEHS panel 2). After excluding windows with no SNPs and sequenced regions shorter than 5-kb, 211 windows were available (reference windows). The presence of resequencing gaps was accounted for in all calculations.

### Haplotype Analysis and TMRCA Calculation

Haplotypes were inferred using PHASE (version 2.1) (Stephens et al. 2001; Stephens and Scheet 2005). Linkage disequilibrium (LD) analyses were performed using Haploview (v. 4.1) (Barrett et al. 2005), and blocks were identified through the confidence interval algorithm implemented in the software (Gabriel et al. 2002). Data for LD analysis were derived from resequencing data. Median-joining network to infer haplotype genealogy was constructed using NETWORK 4.6.1 (Bandelt et al. 1999). Estimate of the time to the most recent common ancestor (TMRCA) was obtained using an ML coalescent method implemented in GENETREE (Griffiths and Tavare 1994, 1995). The method assumes an infinite-site model without recombination; therefore, haplotypes and sites that violate these assumptions need to be removed: In the case of IFI16, we eliminated 1 variant. The mutation rate μ was obtained on the basis of the divergence between human and chimpanzee and under the assumption both that the species separation occurred 6 Ma (Glazko and Nei 2003) and of a generation time of 25 years. Using this μ and θ ML (θ_ML_), we estimated the effective population size parameter (*N*_e_), which resulted equal to 18,000. With these assumptions, the coalescence time, scaled in 2*N*_e_ units, was converted into years. For the coalescence process, 10^6^ simulations were performed. A second TMRCA estimate was obtained by application of a method (Evans et al. 2005) that calculates the average pairwise difference between all chromosomes and the MRCA: This value was converted into years on the basis of mutation rate retrieved as above. The standard deviation (SD) for this estimate was calculated as described previously (Thomson et al. 2000). Using this method, the TMRCA was calculated for the *IFI16-*5 kb region and for 5-kb windows from NIEHS genes (one window/gene). In particular, windows were randomly selected with the only requirement that they did not contain any resequencing gap. After discarding X-linked loci, and windows containing no SNPs, 200 windows were used for TMRCA calculation.

## Results

### IFI16 and AIM2 Evolved Adaptively in Primates

To analyze the evolutionary history of ALR genes (*IFI16*, *AIM2*, *MNDA*, and *PYHIN1*) in primates, we obtained coding sequence information for 16 species from public databases or by sequencing (see Materials and Methods). In humans, the *IFI16* gene carries a polymorphic segmental duplication of exon 7, with the sequence of the two exons being identical (see later). We applied a PCR-based approach to determine the status of the exon 7 segmental duplication in different nonhuman primates, namely eight chimpanzees, one gorilla, and one orangutan. Results indicated the presence of the duplicated exon in gorilla and orangutan, whereas all chimpanzee samples carried a single copy of exon 7. Sequencing of the *IFI16* mRNA in *M. fascicularis* and *C. aethiops* also showed the presence of a single copy of exon 7. Thus, the nonduplicated gene sequence was used for multiple species alignment.

We calculated the average nonsynonymous substitution/synonymous substitution rate (d*N*/d*S*, also referred to as ω) for the four ALR genes using the single-likelihood ancestor counting (SLAC) method (Kosakovsky Pond and Frost 2005). This analysis indicated that *IFI16* might evolve under positive selection, as the average d*N*/d*S* was higher than 1, whereas *AIM2*, *MNDA*, and *PHYIN1* showed d*N*/d*S* values < 1 (supplementary table S3, Supplementary Material online), suggesting a role for purifying selection.

Nonetheless, positive selection might act on a few sites within a gene, which is elsewhere selectively constrained. To test this possibility, and to gain further insight into the evolutionary history of *IFI16*, we applied ML analyses implemented in the PAML package (Yang 1997, 2007). Because recombination might yield false-positive results when testing for selection (Anisimova et al. 2003), we first screened the alignments for evidence of recombination using GARD (Kosakovsky Pond et al. 2006); this analysis underscored no recombination breakpoint in any alignment. Thus, we used the codeml program to compare models of gene evolution that allow (NSsite models M2a and M8) or disallow (NSsite models M1a and M7) a class of codons to evolve with d*N*/d*S* > 1. For *IFI16* and *AIM2,* the two neutral models were rejected in favor of the positive selection models; this result was confirmed using different codon frequency models (F61 and F3x4) (table 1).

**Table 1:** LRT Statistics for Models of Variable Selective Pressure among Sites (F3X4 and F61 Models of Codon Frequency)

Gene (Number of Codons)	df	−2Δln *L*	*P*	Percentage of Sites (Average d*N*/d*S*)
*AIM2* (347)				
F3X4				
M1a vs. M2a	2	13.13	0.001	18.94 (2.4)
M7 vs. M8	2	15.54	0.0004	17.2 (2.5)
F61				
M1a vs. M2a	2	7.80	0.02	22.2 (2.0)
M7 vs. M8	2	9.16	0.01	22.0 (2.0)
*IFI16* (731)				
F3X4				
M1a vs. M2a	2	39.32971	2.88 × 10^−09^	14.8 (3.9)
M7 vs. M8	2	39.04452	3.32 × 10^−09^	17.8 (3.6)
F61				
M1a vs. M2a	2	32.75	7.74 × 10^−8^	22.74 (2.9)
M7 vs. M8	2	32.59	8.37 × 10^−8^	29.46 (2.6)
*MNDA* (414)				
F3X4				
M1a vs. M2a	2	6.30	0.04	8.2 (3.0)
M7 vs. M8	2	6.31	0.04	10.3 (2.7)
F61				
M1a vs. M2a	2	3.33	0.19	—
M7 vs. M8	2	2.64	0.27	—
*PYHIN1* (492)				
F3X4				
M1a vs. M2a	2	5.11	0.08	—
M7 vs. M8	2	5.12	0.08	—
F61				
M1a vs. M2a	2	2.88	0.24	—
M7 vs. M8	2	2.98	0.22	—

Note.—M1a is a nearly neutral model that assumes one ω class between 0 and 1 and one class with ω = 1; M2a (positive selection model) is the same as M1a plus an extra class of ω > 1. M7 (null model) assumes that 0 < ω < 1 is beta distributed among sites in 10 classes; M8 (selection model) has an extra class with ω ≥ 1; 2Δln *L*: twice the difference of the natural logs of the maximum likelihood of the models being compared; *P*: *P* value of rejecting the neutral models (M1a or M7) in favor of the positive selection model (M2a or M8); percentage of sites (average d*N*/d*S*): estimated percentage of sites evolving under positive selection by M8 (d*N*/d*S* for these codons).

To identify specific sites subject to positive selection, we applied two methods, the BEB analysis (with a cutoff of 0.90) from M8 and the MEME (with the default cutoff of 0.1): Only sites detected using both methods were considered, and these are shown in figure 1.

We next mapped positively selected sites in *IFI16* and *AIM2* onto protein domain or three-dimensional structures.

Three positively selected sites in AIM2 map to the HIN domain (fig. 1), and one is located in the PYD (helix α4). The crystal structure of the HIN domain has been solved, and it displays two oligonucleotide/oligosaccharide-binding (OB) folds forming the DNA-binding surface (Jin et al. 2012). Two of the positively selected residues in AIM2 are located in the region responsible for DNA binding, one in the OB1 region and one in the OB2 region (Jin et al. 2012) (fig. 1). In particular, 166T is immediately adjacent to a residue (165F) that, if mutated, strongly reduces DNA binding (Burckstummer et al. 2009). The third positively selected residue is located at the N terminus of the HIN domain, which is not directly involved in DNA binding.

In IFI16, we identified 14 positively selected sites. Three of them are located in the PYD domain; by comparison with the crystal structure of the homologous AIM2 domain (Jin et al. 2013), these residues are predicted to be located in the α1 (2G) and α6 (92P) helices and in the short loop connecting the α2 to the α3 helix (37L) (fig. 1) (Jin et al. 2013). The majority of positively selected sites in IFI16 are clustered in the interdomain regions (separating the two HIN or the PYD from the first HIN) (fig. 1). One of these sites (141G) is located within an accessory nuclear localization signal (referred to as motif-4) (Li et al. 2012). The positively selected 615 position in the second HIN domain could be mapped onto the crystal structure and was found to be located at the DNA-binding interface formed by the four IFI16 monomers (fig. 1) (Jin et al. 2012).

**Fig. 2.—:**
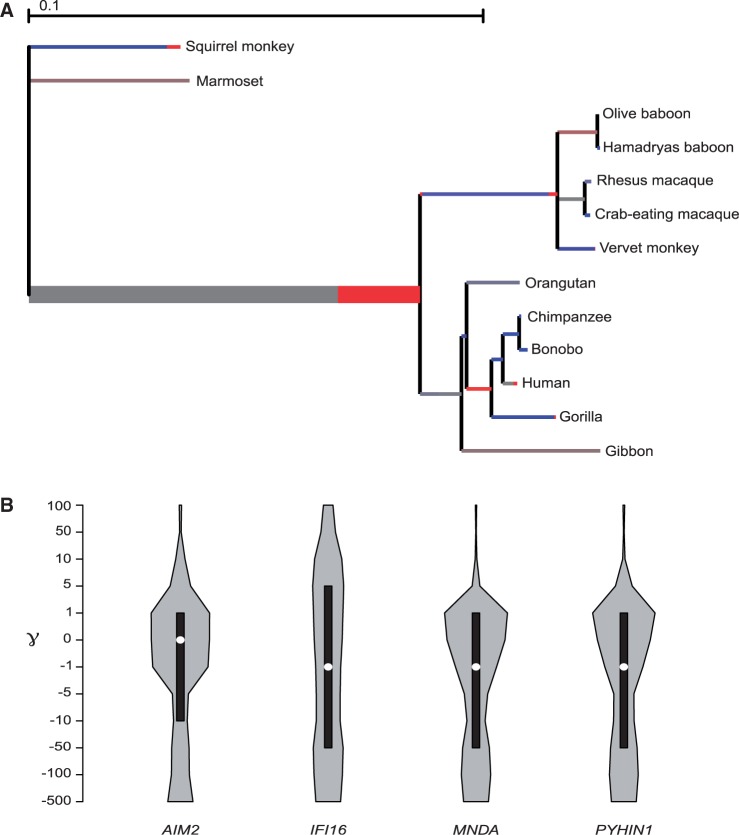
Lineage-specific selection and DFE analysis. (*A*) Branch-site analysis of positive selection in IFI16. Branch lengths are scaled to the expected number of substitutions per nucleotide, and branch colors indicate the strength of selection (ω). Red, positive selection (ω > 5); blue, purifying selection (ω = 0); gray, neutral evolution (ω = 1). The proportion of each color represents the fraction of the sequence undergoing the corresponding class of selection. Thick branches indicate statistical support for evolution under episodic diversifying selection as determined by BS-REL. (*B*) Violin plot of selection coefficients (γ) for ALR genes (median, white dot; interquartile range, black bar). Selection coefficients are classified as strongly beneficial (100, 50), moderately beneficial (10, 5), weakly beneficial (1), neutral (0), weakly deleterious (−1), moderately deleterious (−5, −10), strongly deleterious (−50, −100), and inviable (−500).

To explore possible variations in selective pressure among different lineages for *IFI16* and *AIM2*, we next tested whether a model that allows d*N*/d*S* to vary along branches (model M1) had significant better fit to the data than a model that assume one same d*N*/d*S* across the entire phylogeny (model M0) (Yang and Nielsen 1998). This was indeed the case for *IFI16* (table 2), indicating that different primates experienced variable levels of selective pressure at this gene. We thus used the BS-REL method (Kosakovsky Pond et al. 2011) to identify lineages on which a subset of sites has evolved under positive selection. BS-REL makes no a priori assumption about which lineages are more likely to represent selection targets; the method identified the internal branch leading to catarrhini in *IFI16* (fig. 2). This branch was cross-validated using the branch-site models implemented in PAML (Zhang et al. 2005), which apply a LRT to compare a model (MA) that allows positive selection on one or more lineages (foreground lineages) with a model (MA1) that does not allow such positive selection (table 2). Through BEB analysis, the PAML branch-site model allows identification of specific sites evolving under positive selection in the foreground branches; this procedure is accurate but has low statistical power (Zhang et al. 2005). Because MEME was specifically developed to detect episodic positive selection (in addition to pervasive selection), at least some lineage-specific BEB sites should have been identified by the MEME analysis we performed on the whole phylogeny. Indeed, BEB identified five sites for the catarrhini branch (fig. 1) and two were detected by MEME. All branch-specific sites are located in interdomain regions (fig. 1). Interestingly, the 131K residue is located within one of the two major nuclear localization signals (motif-2) (Li et al. 2012).

**Table 2:** LRT Statistics for Models of Positive Selection on Specific Branches

Gene	LRT^a^ Model	Foreground Branch^a^	Codon Frequency Model	Degree of Freedom	−2Δln *L*^b^	*P*^c^
AIM2	M0 vs. M1^d^		F3x4	23	31.89	0.10
IFI16	M0 vs. M1^d^		F3x4	22	40,22	0.01
	MA1 vs. MA^e^	Catarrhini	F3x4	1	15.23	9.52 × 10^−5^

^a^Likelihood ratio test (LRT).

^b^2Δln *L*: Twice the difference of the natural logs of the maximum likelihood of the models being compared.

^c^*P* value of rejecting the neutral model in favor of the positive selection model.

^d^M0 and M1 are free-ratio models, which assume all branches to have the same ω (M0) or allow each branch to have its own ω (M1).

^e^MA and MA1 are branch-site models that assume four classes of sites: the MA model allows a proportion of codons to have ω ≥ 1 on the foreground branches (those to be tested for selection), whereas the MA1 model does not.

### Evolution of ALR Genes in the Human Lineage

We next applied a recently developed population genetics–phylogenetics approach to study the evolution of ALR genes in the human lineage. Specifically, we applied the GammaMap program (Wilson et al. 2011) that jointly uses intraspecific variation and interspecific diversity to estimate the DFEs (i.e., selection coefficients, γ) along coding regions. To this aim, we exploited data from the 1000 Genomes Pilot project deriving from the low-coverage whole-genome sequencing of 179 individuals with different ancestry: CEU, YRI from Nigeria, and AS (Japanese plus Chinese) (1000 Genomes Project Consortium et al. 2010). The ancestral sequence was reconstructed by parsimony from the human, chimpanzee, orangutan and macaque sequences. We first applied GammaMap to obtain the overall distribution of selection coefficients along the four ALR genes. A general preponderance of codons evolving under negative selection (γ < 0) was observed for all genes excluding *AIM2*, which showed most codons to evolve with selection coefficients around neutrality (ranging from −1, weakly deleterious to 1, weakly beneficial). *IFI16* showed the highest proportion of codons with γ > 5 (fig. 2)

GammaMap allows to identify specific codons evolving under positive selection. Herein, we defined positively selected codons as those having a cumulative probability >0.80 of γ ≥ 1. Five such codons were identified in *IFI16* and none in the remaining ALR genes. Two of the *IFI16* sites had previously been identified in the positive selection analysis we conducted on the whole mammalian phylogeny (supplementary table S4, Supplementary Material online). Three of the positively selected sites identified by GammaMap are located in the PYD domain (fig.1, supplementary table S4, Supplementary Material online). Two of them (67K and 70E) are within the α5 helix (Jin et al. 2013) (fig.1); the corresponding residues in the AIM2 PYD are predicted to be highly exposed (Jin et al. 2013).

### Population Genetic Differentiation in Human Populations

We next addressed the role of natural selection in the shaping of genetic diversity at ALR genes in human populations. To this aim, we initially performed an analysis of population genetic differentiation, herein measured as *F*_ST_ (Wright 1950). High-*F*_ST_ values suggest that natural selection drives allele frequencies in distinct populations to differ more than expected on the basis of drift or demography alone. To analyze human population genetic differentiation along the ALR cluster, we exploited two partially independent sets of data. The first set is accounted for by genotype data generated by the 1000 Genomes Pilot project. Using these data, we calculated *F*_ST_ for the three pairwise comparisons (YRI/CEU, AS/CEU, and YRI/AS) (Wright 1950) in sliding windows moving along the genomic region where the four ALR genes are located. Sliding window analyses pose a multiple testing problem that is difficult to correct because of the nonindependence of windows. Moreover, the 1000 Genomes Pilot Project data suffer from a bias in the site frequency spectrum (SFS)*,* with reduced power to detect low-frequency variants (1000 Genomes Project Consortium et al. 2010). To partially account for these limitations, we applied an outlier approach by obtaining *F*_ST_ distributions for the three pairwise comparisons in sliding windows from 2,000 randomly selected human genes. This allowed calculation of the 95th percentile and identification of regions in the ALR gene cluster above this threshold. A complementary set of data, namely the SNP genotypes from the Human Genome Diversity Panel (Li et al. 2008), was also used to analyze population genetic differentiation at the ALR gene cluster. For all HGDP SNPs within the cluster, we obtained *F*_ST_ values among continental groups; these values were compared with the distribution of *F*_ST_ calculated for HGDP variants in the same minor allele frequency (MAF) class. The HGDP panel includes 52 populations distributed worldwide and therefore represents a set of data largely independent from the 1000 Genomes Pilot Project. We thus focused on region of high *F*_ST_ identified using both data sets.

**Fig. 3.—:**
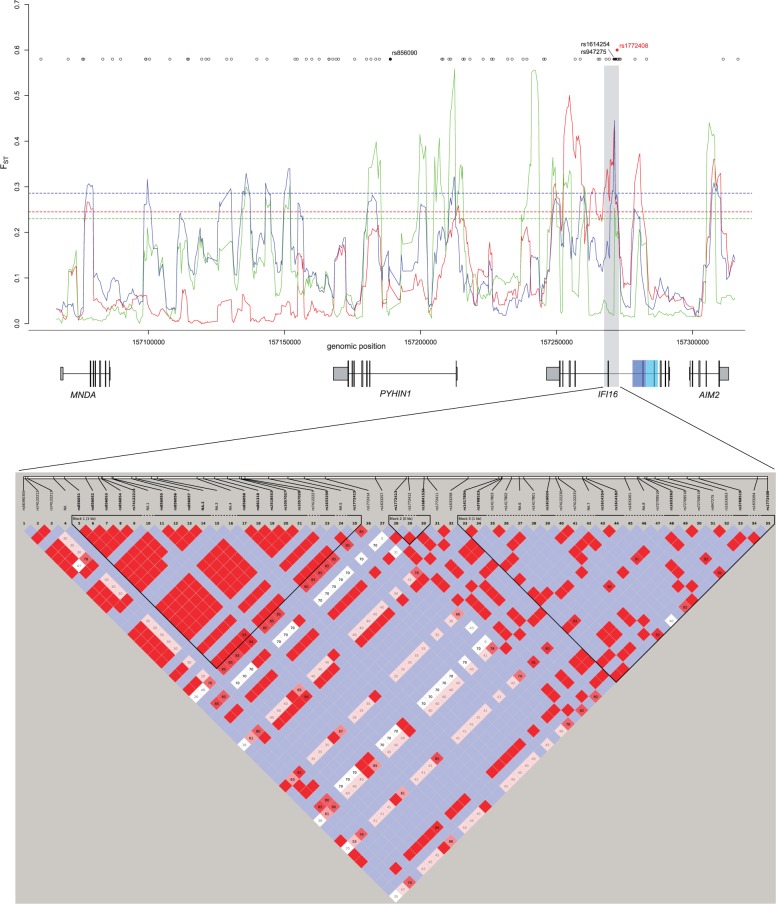
*F*_ST_ analysis of the ALR gene cluster. Data from the 1000 Genomes Pilot Project were used to calculate *F*_ST_ in sliding windows of 20 SNPs moving along the ALR gene cluster (NCBI/hg18, chr1:157063927–157317926) with a step of three SNPs (upper panel). Color codes refer to population comparisons: red, YRI/CEU; blue, YRI/AS; and green, CEU/AS. Horizontal dashed lines represent the 95th percentile in the distribution of *F*_ST_ calculated for sliding windows deriving from 2,000 randomly selected human genes. SNPs genotyped in the HGDP-CEPH panel are represented as gray circles (no unusual *F*_ST_ value among continental groups) or black circles (*F*_ST_ outliers); a SNP associated to rheumatoid arthritis and celiac disease is reported in red. The resequenced *IFI16* region is shaded in gray. The blue and cyan boxes represent the segmental duplication of exon 7. In the bottom panel, LD analysis for the *IFI16* resequenced region (5 kb) is shown. LD analysis was performed with the Haploview software using resequencing data, and blocks were identified through the implemented confidence interval algorithm (see Materials and Methods). Variants within the first LD block were used for Network and GENETREE analyses.

As shown in figure 3, three variants (rs856090:A>G, rs1614254:T>C, rs947275:T>C) were found to be outliers among HGDP continental groups in *F*_ST_ distribution values (ranks = 0.965, 0.951, and 0.981, respectively). Two of them are within the *IFI16* gene, and they are located in a peak of significantly high *F*_ST_ in all pairwise comparisons (YRI/CEU, YRI/AS, and CEU/AS), as assessed from the 1000 Genomes Pilot project data. Interestingly, susceptibility alleles for rheumatoid arthritis and for celiac disease (rs1772408:T>C) were identified in this region through a genome-wide association study (GWAS) (Zhernakova et al. 2011). Also, the *F*_ST_ outliers flank the exon 7 duplication allele (fig. 3).

### Balancing Selection Maintains Genetic Diversity at the *IFI16* Gene in Human Populations

Given the results above, we decided to focus our attention on the *IFI16* gene region carrying the *F*_ST_ outlier SNPs. Because of the aforementioned SFS bias in the low-coverage 1000 Genomes data (1000 Genomes Project Consortium et al. 2010), we decided to resort to Sanger resequencing, so that further analyses could be performed within the framework of coalescent theory. Thus, we resequenced a 5-kb IFI16 region (*IFI16-*5 kb, fig. 3) encompassing the *F*_ST_ peaks and HGDP outliers in three HapMap populations, namely YRI, CEU, and AS. A PCR-based approach was also applied to these samples to determine allelic status for the exon 7 segmental duplication. The duplicated allele was detected in CEU only with a frequency of 7.5%.

Using Sanger-sequencing data, we first calculated *F*_ST_ for the entire *IFI16-*5 kb region in CEU/AS, CEU/YRI, and AS/YRI comparisons; these values were compared with the distribution of *F*_ST_ calculated for 5-kb windows (hereafter referred to as reference windows) deriving from 238 genes resequenced by the NIEHS Program in the same populations. For the YRI/CEU comparison, an *F*_ST_ of 0.38 was obtained, corresponding to a percentile rank of 0.972 in the distribution of reference windows, confirming high population differentiation at the *IFI16-*5 kb region.

We next calculated θ_W_ (an estimate of the expected per site heterozygosity [Watterson 1975]) and π (the average number of pairwise sequence nucleotide differences between haplotypes [Nei and Li 1979]) and, again, compared the values with the distribution obtained from reference windows. The percentile ranks corresponding to *IFI16-*5 kb region in the distribution of NIEHS gene values indicate that the analyzed region displays high nucleotide diversity in all populations, although the rank of π in CEU did not reach the 95th percentile (table 3).

**Table 3:** Nucleotide Diversity and Neutrality Tests for the Analyzed *IFI16* Region

Population	*N*^a^	*S*^b^	Π (×10^−4^)		Θ_W_ (×10^−4^)		Tajima's *D*		Fu and Li's D*		Fu and Li's F*	
			Value	Rank^c^	Value	Rank^c^	Value (*P*)^d^	Rank^c^	Value (*P*)^d^	Rank^c^	Value (*P*)^d^	Rank^c^
YRI	40	47	28.22	**0.99**	22.70	**0.99**	0.86 (**0.028**)	**0.95**	1.06 (**0.014**)	**0.97**	1.18 (**0.01**)	**0.97**
CEU	40	33	15.31	0.94	15.94	**0.98**	−0.14 (0.44)	0.50	0.90 (0.11)	0.84	0.65 (0.23)	0.74
AS	40	26	21.84	**0.99**	14.01	**0.97**	1.92 (**0.031**)	**0.98**	1.78 (<**0.01**)	**0.99**	2.16 (**<0.01**)	**0.99**

Note.—Significant values are in bold.

^a^Sample size (chromosomes).

^b^Number of segregating sites.

^c^Percentile rank relative to a distribution of 238 5-kb windows from NIEHS genes.

^d^*P* value obtained by coalescent simulations.

To confirm this observation, we applied a multilocus MLHKA test (Wright and Charlesworth 2004) by comparing polymorphism and divergence levels at the *IFI16-*5 kb with 99 randomly selected NIEHS genes resequenced in the same populations (YRI, CEU, and AS). Results, summarized in table 4, indicate that a significant excess of nucleotide diversity versus interspecies divergence is detectable in all populations for the *IFI16* study region.

**Table 4:** MLHKA Test for the *IFI16* Gene Region

Population	MLHKA	
	*k*^a^	*P*
YRI	3.64	5.42 × 10^−4^
CEU	3.63	1.644 × 10^−2^
AS	2.21	5.05 × 10^−3^

^a^Selection parameter (*k* > 1 indicates an excess of polymorphism compared with divergence; *k* < 1 indicates the opposite situation).

Thus, these data indicate that high nucleotide diversity at the *IFI16-*5 kb region may be selectively maintained in human populations. This observation suggests the action of balancing selection, although this latter usually results in low rather than high *F*_ST_ (Charlesworth 2006) (see Discussion).

An effect of balancing selection is a distortion of the SFS toward intermediate frequency alleles. Common neutrality tests based on the SFS include Tajima's *D* (*D*_T_) (Tajima 1989) and Fu and Li's D* and F* (Fu and Li 1993). Because population history beside affecting selective processes also influences the SFS, the significance of neutrality tests was evaluated by performing coalescent simulations with population genetics models that incorporate demographic scenarios (see Materials and Methods). We also applied an empirical comparison by calculating the percentile rank of *D*_T_, F*, and D* in the *IFI16-*5 kb relative to 5-kb reference windows (obtained from 238 NIEHS genes). Neutrality tests indicated departure from neutrality with significantly positive values for all statistics in YRI and AS populations; conversely, no significant values were observed in CEU (table 3). Very similar results were obtained when different demographic models were used for coalescent simulations (Marth et al. 2004; Voight et al. 2005; Gutenkunst et al. 2009) (supplementary table S5, Supplementary Material online).

**Fig. 4.—:**
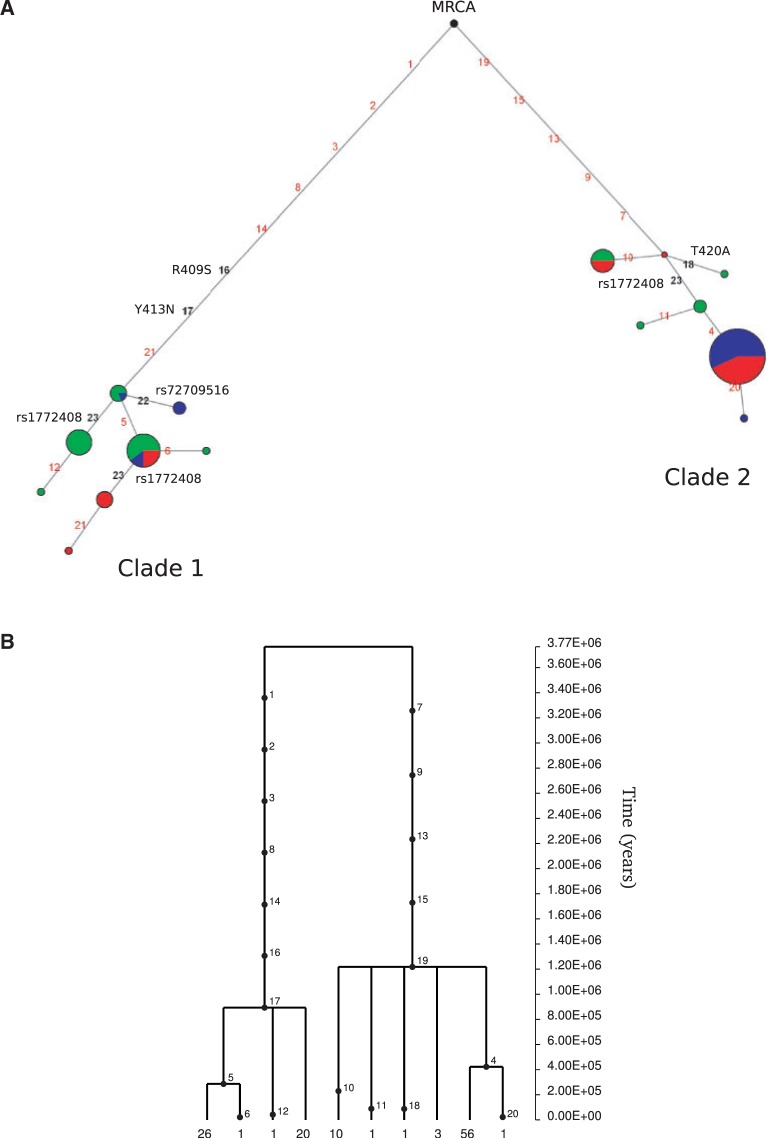
Haplotype analysis of *IFI16*. (*A*) Genealogy of haplotypes in the *IFI16* LD region (1.7-kb region (NCBI/hg18, chr1:157267850–157269530, see text) reconstructed through a median-joining network. Each node represents a different haplotype, with the size of the circle proportional to frequency. Nucleotide differences between haplotypes are indicated on the branches of the network. Color codes are as follows: YRI, green; CEU, blue; and AS, red. The most recent common ancestor (MRCA) is also shown. SNPs mentioned in the text are reported. (*B*) GENETREE for the LD subregion of *IFI16*. Variants are represented as black dots; the absolute frequency of each haplotype is reported.

Further insight into the evolutionary history of a gene region can be gained by inferring haplotype genealogies. In particular, balancing selection is expected to result in two or more major clades with a deep coalescence time. Haplotype genealogies and inference of coalescent times may yield unreliable results in the presence of recombination. Thus, we selected a subregion based on LD; in particular, we used data from a 1.7-kb region (NCBI/hg18 chr1:157267850–157269530) with relatively high LD in all analyzed populations (fig. 3). As it is evident from both the median-joining network (Bandelt et al. 1999) and GENETREE analyses (Griffiths and Tavare 1995) (fig. 4), the haplotype genealogy is split into two major haplogroups (clades 1 and 2) separated by long branches. In line with the *F*_ST_ results, the major clade 2 haplotype is observed in CEU and AS but not in YRI. The time to the most recent common ancestor for the *IFI16* haplotype phylogeny was obtained using GENETREE (Griffiths and Tavare 1995) and amounted to 3.77 Myr (SD: 890 ky).

**Fig. 5.—:**
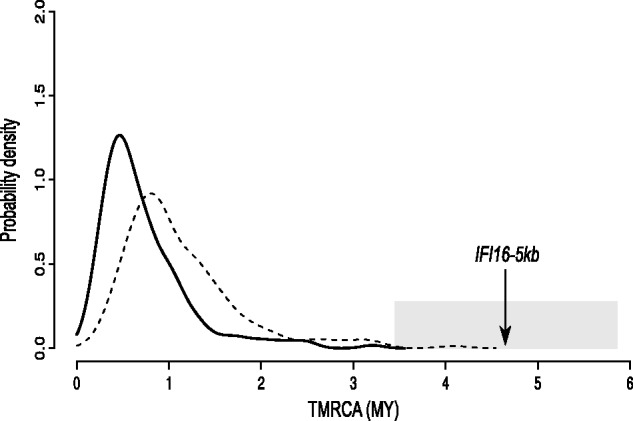
TMRCA estimates for the *IFI16-*5 kb region and for reference windows. Probability density plot of TMRCAs from 5-kb windows deriving from autosomal NIEHS genes (solid line); the upper (TMRCA+SD) estimates are also shown as hatched lines. The TMRCA estimate for the *IFI16-*5 kb region is indicated with upper- and lower bounds (gray shading).

To obtain a second TMRCA estimate for the entire *IFI16-*5 kb region, we applied a previously described method (Evans et al. 2005) that calculates the average pairwise difference between all chromosomes and the MRCA; this value is then converted into years on the basis of the mutation rate (herein calculated on the basis of the number of fixed differences between chimpanzee and humans). Using this approach, we obtained a TMRCA of 4.6 Myr (SD:1.2 Myr) for the *IFI16*-5 kb region. As a comparison, TMRCA estimates were also obtained for 5-kb reference windows using haplotypes from YRI, CEU, and AS subjects, as derived from autosomal NIEHS genes. The mean TMRCA for these windows amounted to 0.76 Myr, in line with previous estimates for human autosomal loci (Garrigan and Hammer 2006)*.* As shown in figure 5, the TMRCA estimate or the *IFI16-*5 kb region is a clear outlier in the TMRCA distribution obtained from reference windows.

Overall, these analyses indicate that the *IFI16* region has been a target of long-standing balancing selection.

To analyze the relationship among the major haplotype clades and the exon duplication allele, as well as the risk variant for autoimmune diseases, two variants located outside the LD region were included in the network: rs1772408:T>C (the GWAS SNP [Zhernakova et al. 2011]) and rs72709516:C>T, which is in full LD with the exon 7 duplication allele (*r*^2 ^= 1 in CEU). Analysis of the haplotype network indicated that, with the exception of few Asian and African haplotypes, rs1772408:T>C separates the two major clades; interestingly, two nonsynonymous variants located in exon 6, rs1057027:A>C (R409S) and rs1057028:A>T (Y413N), are also located on the major branches of the haplotype phylogeny; in line with this observation, the two SNPs are in full LD with the autoimmune risk variant in CEU (*r*^2 ^= 1 in both instances). Finally, a minority of CEU haplotypes in clade 1 are defined by the derived allele at rs72709516:C>T, which is in phase with the nonduplicated exon 7 allele. Analysis of 20 additional HapMap subjects of European descent confirmed full LD between the exon 7 segmental duplication polymorphism and rs72709516, and indicated a MAF of 0.05. This is in line with the reported MAF of 0.041 for rs72709516, as determined by the 1000 Genomes Project in CEU. Thus, either the exon 7 segmental duplication is neutrally evolving or it is subject to very recent/weak selection; its low frequency places it beyond the detection power of most tests based on haplotype homozygosity.

## Discussion

The sensing of foreign genetic material is essential to trigger defensive responses that are important for organism survival to infections. An ever-increasing number of nucleic acid sensors are being identified in mammalian cells, revealing a complex machinery devoted to the detection of the invading pathogen (or of cell damage) and to the transduction of alert signals (Desmet and Ishii 2012). These cellular systems are expected to be engaged in a constant arms race with viruses and other microbial agents (Quintana-Murci and Clark 2013). Genetic conflicts leave signatures on the host genome, and protein regions directly contacting pathogen components are expected to evolve under the strongest diversifying selection. Therefore, evolutionary analyses can be applied both to study the history of host–pathogen interaction and, as recently demonstrated, to identify regions and residues directly involved in viral recognition or, more generally, in antiviral activity (Mitchell et al. 2012). On their side, viruses also evolve products that interfere with pathogen sensing, suggesting that positive selection on host proteins may also result from evolution away from viral recognition.

Results herein indicate that *AIM2* and *IFI16*, the best characterized members of the ALR family, evolved under positive selection in primates. Although only four selected residues were identified in *AIM2*, it should be noted that we defined positively selected sites by the combined use of two methods, BEB and MEME. Although this choice was taken to limit the number of false positive results, we most likely underestimated the number of selected sites, as these methods have different power to detect episodic and pervasive selection (Murrell et al. 2012). Three positively selected sites in AIM2 (166T and 333T) and in IFI16 (615V) are located at HIN domain/DNA-binding interface (fig. 1); one of them (166T) is adjacent to an AIM2 residue that has central importance in DNA binding (Burckstummer et al. 2009). Although AIM2 and IFI16 have been reported to bind dsDNA of both viral and bacterial origin, irrespective of GC content or sequence composition (Fernandes-Alnemri et al. 2009; Hornung et al. 2009; Unterholzner et al. 2010), these results suggest that positively selected residues in the HIN domains evolved to modulate recognition of specific substrates. Interestingly, recent evidence has indicated that, in addition to dsDNA, IFI16 can detect stem-rich secondary structures in ssDNA, which are produced during the replication cycle of lentiviruses (Jakobsen et al. 2013). These observations and the extreme plasticity of the ALR cluster in mammals (Brunette et al. 2012) indicate that diversification at ALR genes is evolutionary advantageous and possibly confers wider specificity in foreign nucleic acid recognition.

Overall, among ALR genes, *IFI16* was found to be the target of the strongest diversifying selection, also showing lineage-specific selection in catarrhini and humans.

Several positively selected sites in IFI16 are located in the PYD domain, which is found in all ALR proteins and in other molecules including PYCARD (ASC). Analysis of PYD-containing proteins indicated that the α3 helix is directly involved in PYD–PYD interactions, whereas the α6 helix is the most variable in length and sequence (Jin et al. 2013); the α5 helix comprises many basic residues, some of which are highly exposed at the protein surface. Of the three positively selected sites, we identified in the analysis on the whole phylogeny, one immediately flanks the α3 and another is located within the α6 helices; two of the human-specific selected sites are located in the α5 helix. Overall, these results suggest that diversity at these sites might modulate association with cellular or viral components. Although IFI16 mainly signals through STING, during Kaposi sarcoma-associated herpesvirus infection, it binds PYCARD through the PYD domain (Kerur et al. 2011); this same domain was shown to directly bind BRCA1, which also shows evidence of positive selection in primates (Pavlicek et al. 2004). Also, viral proteins might have evolved to bind IFI16 through either the PYD or other protein domains. Interestingly, the pUL83 tegument protein encoded by human cytomegalovirus (HCMV), a human-specific pathogen and a herpesvirus family member, interacts with the IFI16 PYD domain and blocks its oligomerization upon DNA sensing (Li et al. 2012). pUL83 is a central HCMV mediator of immune evasion and is predicted to establish extensive contacts with the IFI16 PYD domain. Likewise, the ICP0 protein of herpes simplex virus (HSV-1) has been reported to directly bind IFI16 and to target it for degradation (Orzalli et al. 2012). Unfortunately, the molecular details of the ICP0-IFI16 interaction are presently unknown. In general, IFI16 is thought to play a central role in the immune response to herpesviruses; this is likely achieved through the nuclear localization of this sensor, as the viral genome is protected in the cytoplasm by the capsid, but becomes exposed in the nucleus. In line with this view, HSV-1 eludes the surveillance of IFI16 mutants with cytoplasmic localization (Li et al. 2012). IFI16 displays a multipartite nuclear localization signal and its nuclear translocation is regulated by acetylation and phosphorylation (Li et al. 2012), suggesting that IFI16 localization is finely tuned, possibly in a cell-type and stimulus-dependent manner (Veeranki and Choubey 2012). We found one site positively selected in the catarrhini lineage to be located in one of the two motifs that play a nonredundant and essential role in determining IFI16 nuclear localization. One additional site subject to diversifying selection in the whole phylogeny maps to one accessory nuclear localization signal. It will be interesting to determine whether the selective pressure exerted on these motifs is related to specific viral interactors, to coevolution with cellular cofactors, or is secondary to the appearance and spread of viral species with particular cell-type tropism.

Finally, several sites subject to diversifying selection are located in the spacer separating the two IFI16 HIN domains; this region also shows length variation due to the segmental duplication of exon 7 and, at least in humans, to alternative splicing (Johnstone et al. 1998). An interesting possibility is that, by altering the structure of the spacer, selected sites determine subtle differences in HIN domain relative orientation, eventually affecting substrate recognition (Johnstone et al. 1998).

The spacer region also carries polymorphisms that might represent balancing selection targets in human populations. Application of different population genetic tests indicated that the region around exon 6 displays elevated nucleotide diversity, an excess of polymorphism relative to divergence, and a shift in the SFS toward intermediate-frequency alleles. In line with these findings, haplotype analysis indicated the presence of two clades separated by long branches with a deep TMRCA. Overall, these features represent strong molecular signatures of long-term balancing selection. Notably, when relatively constant in time and space, balancing selection may also result in low population genetic differentiation (Charlesworth 2006). Conversely, our data indicate that the *IFI16* region displays unusually high *F*_ST_ values. The possible explanations for these observations are manifold and depend on the underlying reason for the maintenance of the balanced polymorphism/haplotype. Balancing selection may result from different effects, including variable environmental conditions and frequency-dependent selection (Charlesworth 2006). Because of the dynamic nature of these processes, distinct populations may experience variable pressures and, consequently, different relative frequencies of the selected allele(s), resulting in high differentiation. Also, it should be noted that the sliding-window *F*_ST_ analysis revealed other peaks of high genetic differentiation. We focused on the *IFI16* region, as it was detected by both the 1000 Genomes and by the HGDP genotype data, and because the region carries an autoimmune disease susceptibility variant. Nonetheless, we do not imply that all other ALR gene regions are neutrally evolving in human populations.

Analysis of the *IFI16* haplotype phylogeny suggested that the exon 7 segmental duplication polymorphism does not represent a balancing selection target; in line with the estimated TMRCA, the presence of the duplicated allele in other nonhuman primates (orangutan and gorilla) most likely results from inherent instability (whereby the duplication undergoes nonallelic homologous recombination) rather than from active maintenance due to selection. Conversely, two nonsynonymous variants (R409S and Y413N) in exon 6 separate the major branches of the haplotype network, as would be expected if they represented the selection targets in human populations. R409S and Y413N polymorphisms affect positions conserved among primates, and in CEU, these variants are in full LD with the autoimmune risk variant (rs1772408:T>C), suggesting that they might represent the causal polymorphisms for RA and celiac disease. Interestingly, the rs1772408:T>C variant displays an opposite risk profile: The ancestral allele predisposes to celiac disease but is protective for RA (Zhernakova et al. 2011). This observation suggests that the balancing selection regime results from antagonistic pleiotropy. This is a situation where one locus is associated with more than one trait, with both beneficial and detrimental effects for fitness. Although variants with opposite risk effects on autoimmune diseases are relatively common (Sirota et al. 2009; Wang et al. 2010), few of these have been demonstrated to be maintained as balanced polymorphisms (Cagliani et al. 2011). This is possibly the result of the weak selective effect of autoimmune diseases, which often become clinically relevant at postreproduction ages (Sironi and Clerici 2010). In this case, although celiac disease presents early in life, the widespread use of gluten-containing foods has likely appeared too recently in human populations to account for the long-standing balancing selection signature we describe herein. Therefore, one possible explanation is that functionally different *IFI16* variants were originally maintained by antagonistic pleiotropy related to immune response against pathogens, with differential susceptibility to autoimmune diseases being a consequence. This hypothesis is in line with recent analyses of genes subject to long-standing balancing selection in humans (Cagliani et al. 2010, 2012; Segurel et al. 2012; Leffler et al. 2013) and with theoretical modelling of host–pathogen arms races (Tellier and Brown 2007).

In summary, our data indicate that, in analogy to other nucleic acid sensors (Fumagalli et al. 2010; Vasseur et al. 2011; Patel et al. 2012; Quintana-Murci and Clark 2013), AIM2 and IFI16 have evolved adaptively in primates; in particular a continuum of selective pressure acting on *IFI16* is observed as the gene also represents a selection target in human populations. We suggest that the underlying scenario is the result of an ancestral and still ongoing host–pathogen arms race and that the maintenance of susceptibility alleles for autoimmune diseases at *IFI16* represents an evolutionary trade-off. Ultimately, our results provide evolutionary and functional information about candidate ALR gene variants that might affect immunologic phenotypes.

## Supplementary Material

Supplementary tables S1–S5 are available at *Genome Biology and Evolution* online (http://www.gbe.oxfordjournals.org/).

Supplementary Data
