# Supplementary material for: Ancient and Recent Selective Pressures Shaped Genetic Diversity at AIM2-Like Nucleic Acid Sensors
Source: Genome Biol Evol. 2014 Mar 28;6(4):830–45. doi: 10.1093/gbe/evu066 (PMC4007548; doi:10.1093/gbe/evu066)
Supplement: Supplementary Data [file supp_6_4_830__index.html]

Ancient and recent selective pressures shaped genetic diversity at AIM2-like nucleic acid sensors — Ancient and Recent Selective Pressures Shaped Genetic Diversity at AIM2-Like Nucleic Acid Sensors — Supplementary Data 

# Ancient and Recent Selective Pressures Shaped Genetic Diversity at AIM2-Like Nucleic Acid Sensors

## Supplementary Data

files

**Files in this Data Supplement:**

- Supplementary Data - pdf file
