# Supplementary material for: Ancient and Recent Selective Pressures Shaped Genetic Diversity at AIM2-Like Nucleic Acid Sensors
Source: Genome Biol Evol. 2014 Mar 28;6(4):830–45. doi: 10.1093/gbe/evu066 (PMC4007548; doi:10.1093/gbe/evu066)
Supplement: Supplementary Data [file supp_evu066_Supplementary.pdf]

Supplementary Information for:

Ancient and recent selective pressures shaped genetic diversity at AIM2-like nucleic acid sensors

Rachele Cagliani, Diego Forni, Mara Biasin, Manuel Comabella, Franca R. Guerini, Stefania Riva, Uberto Pozzoli, Cristina Agliardi, Domenico Caputo, Sunny Malhotra, Xavier Montalban, Nereo Bresolin, Mario Clerici, Manuela Sironi

Supplementary Table S1. Primer sequences.

Supplementary Table S2. List of primate species**.**

Supplementary Table S3. Average dN/dS for ALR genes.

Supplementary Table S4. IFI16 positively selected sites in the human lineage.

Supplementary Table S5. Coalescent simulations using different demographic models.

Supplementary Table S1. List of primer sequences used for the amplification and sequencing of *IFI16* cDNA (primates) and for genomic DNA (human population genetics).

	Primer name	Sequence
RT-PCR amplification and sequencing	Fa	CACTTATGTCTGTAAAGATGGG
	Ra	CATTTCTGGGGTCTCGTATT
	Fb	CAGTGGCCAAATGTCAGGT
	Rb	TTGTGACATTGTCCTGTCC
	Fc	CTGAAGCTGGTCCTAACCAAAC
	Rc	GTGAAGAAACTGCTGGATGGAG
	Fd	GAAACAATGACCCCAAGAGCAT
	Rd	AGGTCACTCTGGGCACTGTCTT
	Fe	ATGACCAGCATAGGCCCAGCT
	Re	CCACTTCCATCTTCCCTGT
	Ff	GACCTAAAGGAGAAGTTCACC
	Rf	AAGCACTCCCAATACCCCATT
Genomic DNA amplification and sequencing	pcr-F1	AGTTACAGGCCTCATCCAGT
	seq-F1a	GAGAGAGACCATGTTTGGAAG
	seq-R1a	CTTCCAAACATGGTCTCTCTC
	seq-R1b	CACCAGTGATGACATAGGAG
	pcr-F2	ACAGGGTCAGCATAGCTCAG
	pcr-R1	ACACTCTGATCACCACTGGC
	seq-F2a	CACTTACTTGGTAATGCCAAG
	seq-F2b	CTTGAACCCAACTCTCCACAG
	seq-F2c	CTCCATAGTTTATGCAGGTG
	pcr-R2	CTGCCACTTATTCACGAGAG

Supplementary Table S2. List of primate species.

Common name	Scientific name
Human	*Homo sapiens*
Chimpanzee	*Pan troglodytes*
Pygmy chimpanzee	*Pan paniscus*
Gorilla	*Gorilla gorilla*
Orangutan	*Pongo pygmaeus*
Olive baboon	*Papio anubis*
Hamadryas baboon	*Papio hamadryas*
Macaque	*Macaca mulatta*
Crab-eating macaque	*Macaca fascicularis*
African green monkey	*Chlorocebus aethiops*
Gibbon	*Nomascus leucogenys*
Marmoset	*Callithrix jacchus*
Bolivian squirrel monkey	*Saimiri boliviensis*
Tarsier	*Tarsius syrichta*
Bushbaby	*Otolemur garnettii*
Mouse lemur	*Microcebus murinus*

Supplementary Table S3. Average dN/dS for ALR genes.

Gene	Average dN/dS (95% confidence intervals)
AIM2	0.682 (0.604, 0.768)
IFI16	1.091 (0.982, 1.207)
MNDA	0.871 (0.783, 0.965)
PYHIN1	0.732 (0.589, 0.898)

Supplementary Table S4. IFI16 positively selected sites in the human lineage.

Codon	Ancestral AA	Human AA	Pr[a]	Other methods	Domain/region
2	Glu	Gly	0.952	MEME and BEB	PYD domain
67	Gln	Lys	0.989	-	PYD domain
70	Lys	Glu	0.990	-	PYD domain
203	Gly	Arg	0.898	-	HIN domain
464	Glu	Gly	0.845	BEB	Region separating the two HIN domains

[a] Posterior probability of $\gamma > 1$ as detected by gammaMap.

Supplementary Table S5. Summary statistics p values for the analyzed *IFI16* region obtained using different demographic models.

	**Marth et al.**			**Voight et al.**			**Gutenkunst et al.**		
**Pop** [a]	D_T [b]	D* [c]	F* [d]	D_T [b]	D* [c]	F* [d]	D_T [b]	D* [c]	F* [d]
YRI	0.052	0.068	0.045	0.135	0.072	0.069	0.085	0.071	0.049
EU	0.262	0.113	0.165	0.432	0.186	0.315	0.218	0.040	0.066
AS	<0.01	<0.01	<0.01	0.075	<0.01	<0.01	<0.01	<0.01	<0.01

[a] population;
[b] Tajima's D;
[c] Fu and Li's D*;
[d] Fu and Li's F*;

Marth GT, Czabarka E, Murvai J, Sherry ST. 2004. The allele frequency spectrum in genome-wide human variation data reveals signals of differential demographic history in three large world populations. Genetics 166:351-372.

Voight BF, Adams AM, Frisse LA, Qian Y, Hudson RR, Di Rienzo A. 2005. Interrogating multiple aspects of variation in a full resequencing data set to infer human population size changes. Proc. Natl. Acad. Sci. U. S. A. 102:18508-18513.

Gutenkunst RN, Hernandez RD, Williamson SH, Bustamante CD. 2009. Inferring the joint demographic history of multiple populations from multidimensional SNP frequency data. PLoS Genet. 5:e1000695.
